# Targeted and Untargeted Metabolomics Profiling of Wheat Reveals Amino Acids Increase Resistance to *Fusarium* Head Blight

**DOI:** 10.3389/fpls.2021.762605

**Published:** 2021-11-19

**Authors:** Peiying Zhao, Shubo Gu, Chao Han, Yaru Lu, Chunyang Ma, Jichun Tian, Jianjie Bi, Zhiying Deng, Qunqing Wang, Qian Xu

**Affiliations:** ^1^College of Agronomy, Shandong Agricultural University, Tai'an, China; ^2^Shandong Province Key Laboratory of Agricultural Microbiology, Department of Plant Pathology, College of Plant Protection, Shandong Agricultural University, Tai'an, China; ^3^State Key Laboratory of Crop Biology, Shandong Agricultural University, Tai'an, China

**Keywords:** *Fusarium* head blight, metabolomics, wheat, amino acids, disease resistance

## Abstract

*Fusarium* head blight (FHB), a notorious plant disease caused by *Fusarium graminearum (F. graminearum)*, is severely harmful to wheat production, resulting in a decline in grain quality and yield. In order to develop novel control strategies, metabolomics has been increasingly used to characterize more comprehensive profiles of the mechanisms of underlying plant-pathogen interactions. In this research, untargeted and targeted metabolomics were used to analyze the metabolite differences between two wheat varieties, the resistant genotype Sumai 3 and the susceptible genotype Shannong 20, after *F. graminearum* inoculation. The untargeted metabolomics results showed that differential amino acid metabolic pathways existed in Sumai 3 and Shannong 20 after *F. graminearum* infection. Additionally, some of the amino acid contents changed greatly in different cultivars when infected with *F. graminearum*. Exogenous application of amino acids and *F. graminearum* inoculation assay showed that proline (Pro) and alanine (Ala) increased wheat resistance to FHB, while cysteine (Cys) aggravated the susceptibility. This study provides an initial insight into the metabolite differences of two wheat cultivars under the stress of *F. graminearum*. Moreover, the method of optimization metabolite extraction presents an effective and feasible strategy to explore the understanding of the mechanisms involved in the FHB resistance.

## Introduction

*Fusarium* Head Blight (FHB), which causes a critical yield loss and decline in grain quality, is one of the most destructive wheat diseases in the world (Bai et al., 2018). This serious cereal disease is mainly caused by the pathogenic fungus *Fusarium graminearum* (Ma et al., 2019). Breeding resistant wheat varieties is an effective method to resist FHB (Buerstmayr et al., 2020). Previous studies employed molecular biology techniques such as QTL (Quantitative Trait Locus) to determine the resistance mechanism of FHB (Liu et al., 2006; Cai, 2016). At present, seven QTL genes (*Fhb1–Fhb7*) have been identified in wheat (Liu et al., 2006; Cai, 2016; Wang et al., 2020). *Fhb1* was the first obtained gene from Sumai 3, which is a strong and stable wheat variety resistant to *F. graminearum*, and it has been applied to the breeding of wheat varieties resistant to FHB (Liu et al., 2006; Buerstmayr et al., 2009; Rawat et al., 2016). Besides this, the *Fhb7* gene has been studied and cloned, which provides a bright prospect for the development of wheat head blight resistance (He et al., 2018; Wang et al., 2020). In the interaction between wheat and *F. graminearum* (Dweba et al., 2017), plants use complex chemical defense mechanisms involved in metabolic adaptations (Feussner and Polle, 2015; Arbona and Gomez-Cadenas, 2016). Advances in analytical chemistry and high-throughput sequencing were used to acquire extensive metabolic profiles during plant-pathogen (Beccari et al., 2019; Chen et al., 2019). A more comprehensive understanding of the mechanisms of underlying plant-pathogen interactions is needed, in order to develop novel, better and safer control strategies.

The metabolome is the last frontier mediating the communication between the genome and the phenotype of an organism, and now it becomes a potential tool for elucidating host biochemical responses to biotic stress, identifying candidate disease resistance genes, and validating gene function (Kumar et al., 2017; Chen et al., 2019). Most metabolomics studies are based on gas chromatography (GC) or liquid chromatography (LC) coupled with Mass Spectrometry (GC-MS or LC-MS) (Patti et al., 2012; Cajka et al., 2017). The LC-MS system is used to identify the metabolic differences of barley genotypes resistant to FHB, indicating that phenylpropanes, terpenoids, flavonoids, and fatty acids are the main resistance compounds to barley FHB (Kumaraswamy et al., 2011a,b). Furthermore, the metabolomics approach used to identify hydroxycinnamic acid amides (HCAAs), such as coumaroylagmatine and coumaroylputrescine, were the highest fold change RR metabolites in the rachis of a wheat resistant near-isogenic line (NIL-R) under *F. graminearum* infection (Kage et al., 2017). In addition to the secondary metabolites, the direct involvement of plants' primary metabolites such as amino acids not be denied in plant-pathogen interaction (Hildebrandt et al., 2015). The amino acid levels of wheat are significantly increased by deoxynivalenol (DON) treatment, most likely due to an active defense response of the plant triggered by DON alone (Warth et al., 2015). This suggests that pathogenic infection has been found to bring many changes in amino acid metabolism expression (Pratelli and Pilot, 2014). Besides, amino acids play pivotal roles in signaling processes (Fagard et al., 2014). The final outcome may be strengthening plant defenses to effectively resist pathogenic attack before infection (Tegeder, 2014). However, metabolites may hold further secrets pursuant to wheat FHB resistance which remain to be investigated.

Shannong 20 is a new wheat variety cultivated in China with resistance to multiple threats and a high yield, but it shows high susceptibility to FHB (Li et al., 2014). Thus, this study used a new optimized LC-MS method, investigating the metabolites of Shannong 20 and Sumai 3, two wheat genotypes with different FHB resistance levels, to clarify the resistance mechanism of wheat to FHB increase.

## Materials and Methods

### Plant Materials and Inoculation Assay

Sumai 3 and Shannong 20 were used as wheat cultivars in this study and provided by Professor Jichun Tian, planted in the greenhouse of Shandong Agricultural University (Taian, China).

*F. graminearum* strain PH-1 was grown on Potato Dextrose Agar (PDA) medium at 25°C for 3 days. A total of 14 fungal plugs were transferred into a 400 mL conical flask with 200 mL carboxymethyl cellulose (CMC) liquid medium, and cultured with shaking at 220 rpm at 25°C for 3 days (Hou et al., 2002). Then conidia were harvested and resuspended with sterile water to the concentration of 1–5 × 10^5^/mL. Each spike was inoculated with 2 florets (each floret was inoculated with 10 μL conidia suspension), as per the protocol described in Feng et al. (2018). In total, 10 biological replicates were performed for each wheat variety. Resistant genotype (Sumai 3) and susceptible genotype (Shannong 20) spike samples were collected at 0, 3, 6, and 9 days post-inoculation (dpi), labeled as R-0, R-3, R-6, and R-9, and S-0, S-3, S-6, and S-9; each treatment was repeated six times. The spikes were processed with liquid nitrogen freezing and then transferred to a −80°C refrigerator before detection of the metabolome.

### Metabolite Extraction

All chemicals and solvents were analytical or HPLC grade. Water, methanol, acetonitrile, formic acid, and acetic acid were purchased from CNW Technologies GmbH (Düsseldorf, Germany).

The method of metabolite extraction was optimized by referring to previous methods (Guo et al., 2015) for three factors, including extraction solvent, extraction method, and extraction time, with three levels for each factor (Table 1). Due to the multi-factor and multi-level experiment, this experiment adopted L9(3^4^) orthogonal experiments and univariate analysis to assess the optimization of metabolites extraction methods and make variance analysis on the orthogonal test results (Martin et al., 2014). The treatment combination was designed by referring to the L9(3^4^) orthogonal test table (Supplementary Table 1). To obtain sufficient compounds, we used a new generation Orbitrap instrument (Q-Exactive hybrid quadrupole-Orbitrap mass spectrometer) with high-performance quadrupole precursor selection and high-resolution accurate-mass Orbitrap detection, combined with UPLC instruments, providing support for obtaining more compound data extraction of metabolites. The metabolite peaks of each combination method were extracted by UPLC-QE-MS, and variance analysis was conducted on the data to screen out the optimal level combination.

**Table 1:** Factors for optimizing extraction method of sample pretreatment.

**Level**	**Factor**		
	**Extraction solvent**	**Extraction method**	**Extraction time (min)**
1	80%Acetonitrile (0.1%FA)	Shock	10
2	80%Acetonitrile (0.1%HAc)	Ultrasound	15
3	Methanol:Acetonitrile:H_2_O(2:2:1)	Stand still	30

Each of the frozen wheat spike samples was milled to a fine powder for 1 min using a ball mill (GT200 Grinder, China) with liquid nitrogen pre-cooled 10 mL stainless steel vessels and a 9-mm stainless steel ball. The wheat samples (100 ± 2 mg frozen weight) were extracted with 1 mL extraction solution methanol: acetonitrile: water (2:2:1, v/v/v) in 2 mL Eppendorf tubes. We extracted all homogenates via ultrasonic extraction for 10 min and centrifugation at 13,000 rpm for 5 min. We then transferred 700 μL of each of the supernatants into the new centrifuge tube and evaporated all fluid with a quick spin concentrator. Dried metabolite pellets were resuspended by 100 μL methanol and filtered through a 0.22 μm filter membrane, and then could be used in UPLC-QE-MS detection. Quality control (QC) samples were prepared by mixing aliquots of all samples to be a pooled sample.

### UPLC-QE-MS Analysis and Data Preprocessing

A UPLC system (Thermo Fisher Scientific, Bremen, Germany) was used for the separation of metabolites. To monitor the stability and repeatability of the instrument analysis, the QCs were injected at regular intervals (every 10 samples) throughout the analytical queue (Zhang et al., 2019). The column temperature was 35°C, and the flow rate was 0.3 mL/min. The eluents were eluent A (0.04% acetic acid in water) and B (0.04% acetic acid in acetonitrile). The solvent gradient was set as follows: 90% A, 0.2 min; 10–0% A, 6 min; 10% A, 8.0 min; 10–90% A, 8.1 min; 90% A, 10 min (Fiehn et al., 2007).

Detection of compounds was performed using Q-Exactive quadrupole-Orbitrap mass spectrometer equipped with heated electrospray ionization (ESI) source (Thermo Fisher Scientific, Bremen, Germany) operating in data-dependent acquisition (DDA) mode. In positive polarity mode, spray voltage was 3.8 kV, capillary temperature 350°C, sheath gas flow rate 35 arb and aux gas flow rate 10 arb, resolution 17,500, microsweep 1, AGC target 2e5, normalized collision energy was 30. In negative polarity mode, the voltage was 2.9 kV, capillary temperature 350°C, sheath gas flow rate 40 arb, aux gas flow rate 10 arb, resolution 17,500, microsweep 1, AGC target 2e5, and the normalized collision energy was 30.

Data obtained on the UPLC-QE-MS should be converted into mzML format by ProteoWizard software (Holman et al., 2014). The transformed data were processed by XCMS (https://xcmsonline.scripps.edu/) (Smith et al., 2006), such as peak extraction, peak alignment, normalization, etc., to obtain the two-dimensional data matrix of mass/charge ratio (m/z), retention time, similarity, and peak area, and to build a multidimensional data set. The missing data were supplemented by the minimum value complement method, and the data results were de-noised by the quad method to finally determine the positive and negative ion phase variables that met the requirements. Normalization of the peak area was carried out on the data for subsequent analysis.

The software MetDNA (Metabolite identification and Dysregulated Network Analysis) (http://metdna.zhulab.cn/) and SIMCA (v. 13.0, 2011, Umetrics, Umea, Sweden) were used for the large-scale identification of metabolites and multivariate data analysis from LC-MS/MS data sets. Principal component analysis (PCA) and partial least-squares discriminant analysis (PLS-DA) were used to analyze the credibility of the mass spectrum data. The differences of metabolites were analyzed according to the VIP value of PLS-DA model (VIP > 1), and the P-value of Student's *t*-test (*P*-value <0.05) of the peak area of metabolites among different wheat varieties, and the ploidy changes of the peak area.

### Absolute Quantitative Analysis of Amino Acids

The absolute quantification of amino acids (histidine, serine, threonine, arginine, alanine, cysteine, methionine, and proline) was performed by UPLC-QqQ-MS targeted metabolomics. Absolute quantification of amino acids was used by the external standard method, the regression equation developed by plotting different concentrations of pure amino acids (Sigma-Aldrich® Inc., MO, USA) (Matuszewski et al., 2003). Student's *t*-test was used to analyze the significant difference in amino acid content between the two varieties.

The wheat samples (100 ± 2 mg frozen weight), like the untargeted metabolomics samples, were extracted with 1.5 mL sterile water. We extracted all homogenates via ultrasonic extraction at room temperature for 10 min and then centrifuged for 5 min at 13,000 rpm. The supernatant was transferred to a new centrifugal tube, and the amino acids could be detected after filtration by 0.22 μm membrane.

A UPLC system (Thermo Fisher Scientific Inc., Waltham, MA, USA) was used for the separation of metabolites. The column temperature was 35°C, and the flow rate was 0.3 mL/min. The eluents were eluent A (0.1% acetic acid in water) and B (acetonitrile). The solvent gradient was set as follows: 90% A, 0.2 min; 10–90% A, 6 min; 10% A, 8.0 min; 10–90% A, 8.1 min; 90% A, 10 min. Detection of compounds was performed using TSQ Quantive mass spectrometer (Thermo Fisher Scientific Inc., Waltham, MA, USA) operating in the multiple reaction monitoring (MRM) mode, the Quantitive mass spectrometer was operated in positive polarity mode with a spray voltage of 3.8 kV, capillary temperature of 350°C, sheath gas flow rate of 40 arb and aux gas flow rate of 10 arb, microsweep 1, and AGC target 2e5.

### Exogenous Application Amino Acid and Inoculation *F. graminearum* Assay

Inoculation and resistance assessment of in wheat leaves *in vitro*, referring to the method described previously (Chen et al., 2010; Yuan et al., 2011; Kadotani et al., 2016). The experiments were performed in wheat (Shannong 20) at the 3-leaf stage, plants were cultivated in growth chambers under a 16 h light, 28°C/8 h dark, 25°C regime. Wheat leaves were cut to 4 cm in length, and a wound gently poked in the middle position of the paraxial surface of the leaves. Two ends of isolated leaves were dipped into one of the 10 mM amino acids (Gly, Met, Arg, Pro, Ala, and Cys) for 24 h. Water was used in mock treatments as a control. Then, we used *F. graminearum* inoculation on wheat leaves (3 μL), sealed and cultured at 24°C for 3 days. After 3 days, we observed and recorded the area of disease spots on the leaves, and the statistical data were collected to significance analysis by Student's *t*-test

In order to further verify the influence of exogenous amino acids on FHB resistance, and *F. graminearum* inoculation of wheat spikes was used for the identification. In the wheat flowering stage, spraying 10 mM amino acid on wheat flag leaves, after 24 h, we inoculated 10 μL *F. graminearum* in the left and right base florets of the third spikelet. The leaves were then bagged to retain moisture, and the bags discarded after 3 days. After 7 and 14 days, we observed and recorded the number of extended spikelets of FHB, and the statistical data were collected for significance analysis by Student's *t*-test (Kong et al., 2007). Then, pictures were taken and relative virulence was measured by qRT-PCR. The ratios of *F. graminearum* DNA (*GAPDH*, Forward primer: 5′-AAGTTCTACTCTGAGCGTGACCC-3′, reverse primer: 5′-TTGGAGGAAGGACCATCGAC-3′) to wheat DNA (β*-actin*, Forward primer: 5′-GCAAAGAGATCACGGCCCTT-3′, reverse primer: 5′-GCAACTTCCACACTTGAGAGG-3′) were quantified in the infected plants' tissues. All these assays were repeated independently at least three times.

## Results

### Optimization of Metabolites Extraction Methods for Metabolomic Samples

**Figure 1:**
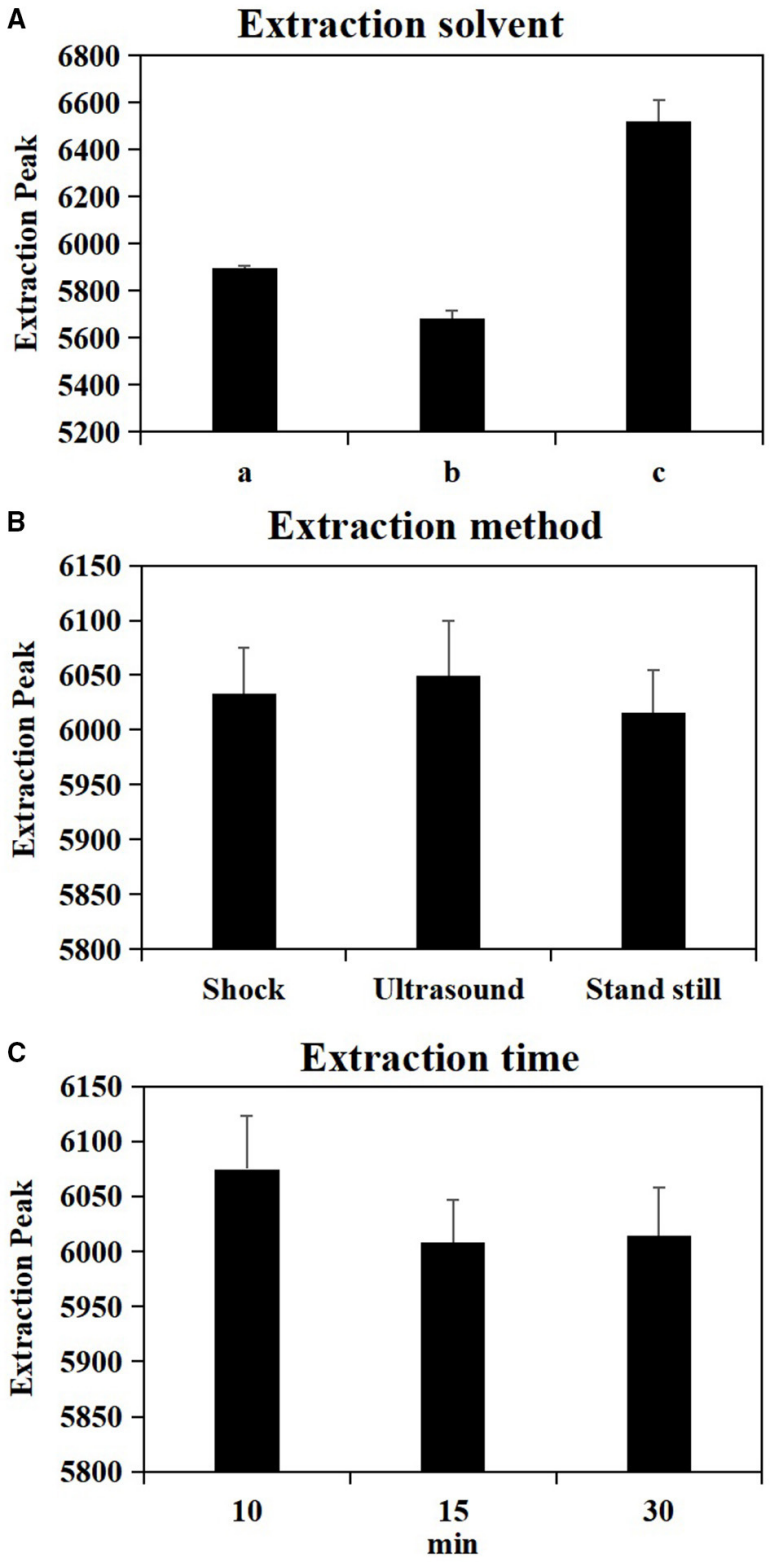
Effect of single factor extraction solvent **(A)**, extraction method **(B)** and extraction time **(C)** on metabolite extraction. a = 80% Acetonitrile (0.1% Formic acid), b = 80% Acetonitrile (0.1% Acetic acid), c = Methanol: Acetonitrile: H_2_O (2:2:1, v/v/v).

As shown in Table 1, the test factors and levels were extraction solvents (A), extraction methods (B), and extraction time (C). Variance analysis on the orthogonal table shows the *R*-value in level extraction solvent was significantly higher in level extraction method and in level extraction time (Supplementary Table 1), suggesting that the factors affecting the number of extraction peaks of plant metabolites are extraction solvents > extraction time > extraction methods. In combination with the results of univariate analysis (Figure 1), the best extraction method of wheat metabolites is confirmed by methanol: acetonitrile: water (2:2:1, v/v/v) and ultrasound for 10 min.

### FHB Resistance Assessment in Sumai 3 and Shannong 20

**Figure 2:**
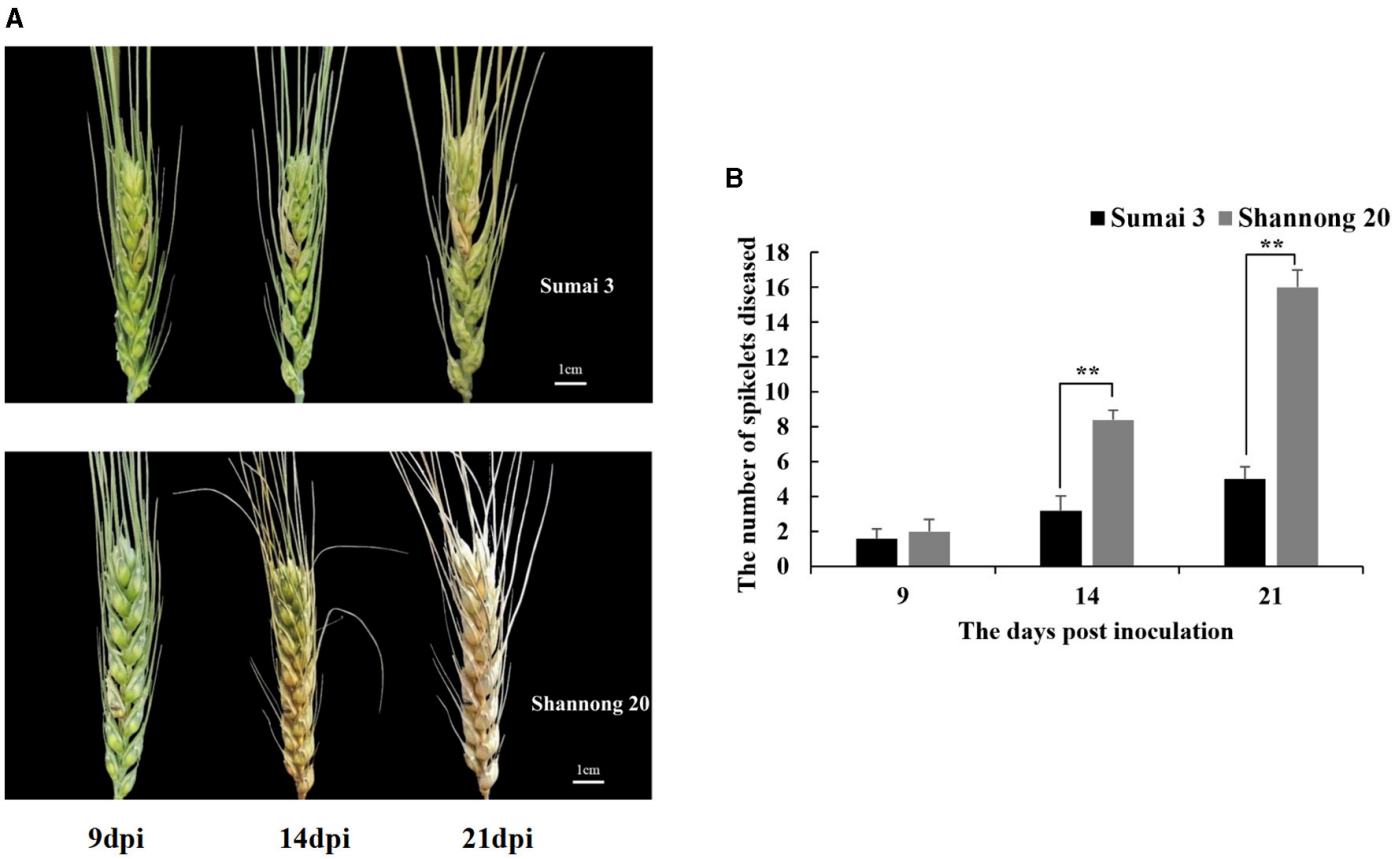
**(A)** *Fusarium* head blight (FHB) symptoms at 9, 14, and 21 days post-inoculation (dpi) in wheat cultivars, Sumai 3 and Shannong 20, inoculated with *F. graminearum*. Shannong 20 is sensitive to FHB and high level of bleaching. **(B)** Number of diseased spikelets in Sumai 3 and Shannong 20. At 14 and 21 dpi, the number of diseased spikelets in Shannong 20 was significantly higher than that in Sumai 3 (*P* value<0.01). Student *t*-test *P*-value: **P value<0.01.

FHB resistance in wheat cultivars Sumai 3 and Shannong 20 was assessed by quantifying the spread of inoculated spikelets to un-inoculated spikelets (Figure 2A). At 9 dpi, the initial symptoms were observed in both Sumai 3 and Shannong 20 inoculated spikelets. Before 9 dpi, there were no significant FHB symptoms differences of Sumai 3 and Shannong 20. Therefore, to explore the potential disease resistance mechanism before FHB symptoms expression, the time before 9 dpi was selected as the emphasis in this study.

The disease progress varied drastically between cultivars (Figure 2B). In all 8 bleached spikelets were observed at 14 dpi in Shannong 20 which increased to the entire spike at 21 dpi. Bleached spikelets were observed in Sumai 3 at 14 dpi, and did not spread to other spikelets until 21 dpi.

### Wheat Metabolite Profile Under FHB Stress

Using UPLC-QE-MS untargeted metabolomics, 5,966, 5,979, 5,987, and 5,997 metabolites were detected in Sumai 3 at 0, 3, 6, and 9 dpi, 5,973, 5,983, 6,014, and 6,033 metabolites were detected in Shannong 20 at 0, 3, 6, and 9 dpi.

**Figure 3:**
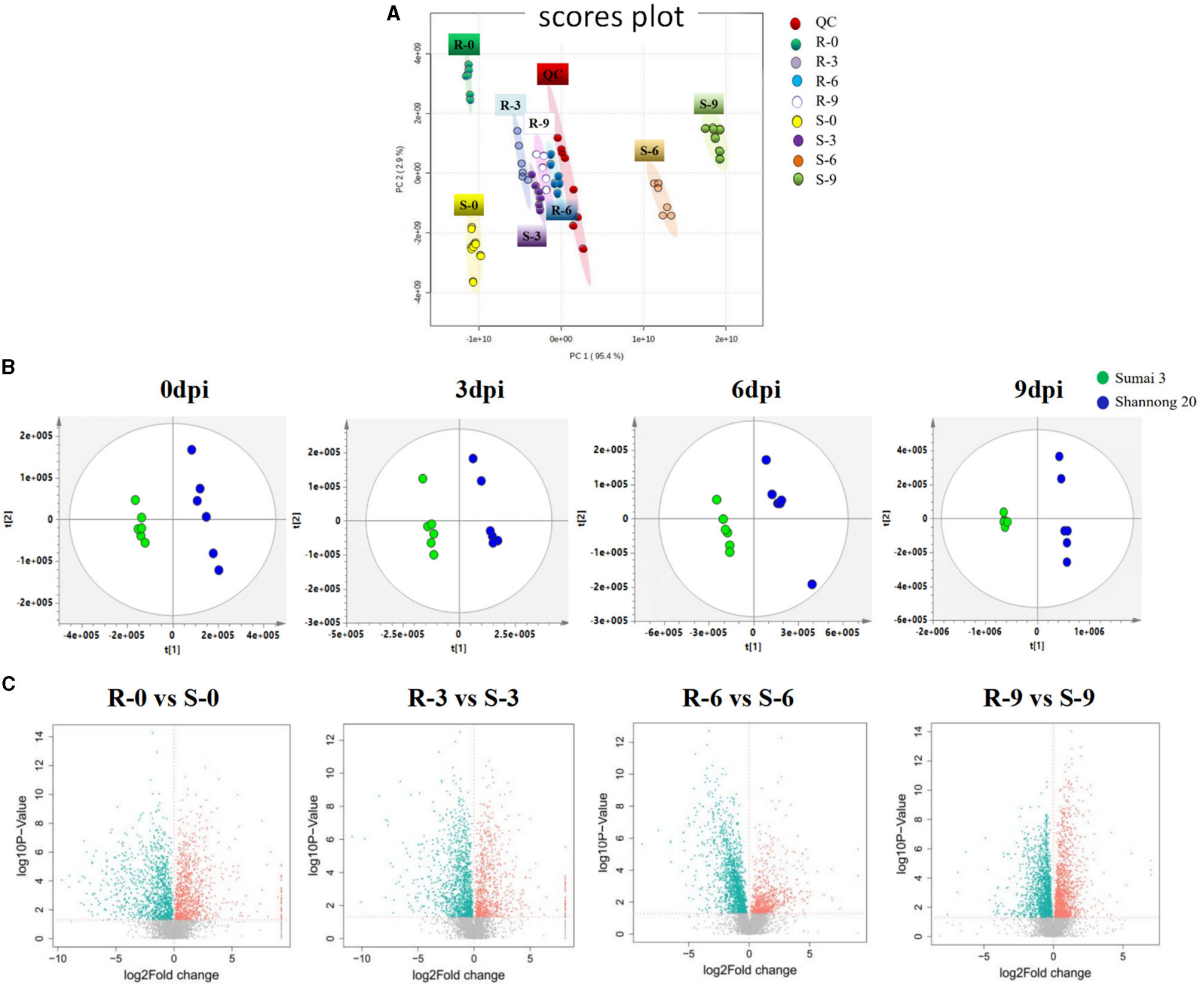
*F. graminearum* inoculation induced a significant metabolic response in spikelets of wheat. **(A)** Principal component analysis (PCA) of metabolic profiles in all samples (six biological replicates). The partial least-squares discriminant analysis (PLS-DA) score plots **(B)** and a volcano plot **(C)** of the comparison between R-0 and S-0, R-3 and S-3, R-6 and S-6, R-9 and S-9, respectively. QC, Quality Control; R-0/3/6/9, resistant genotype (Sumai 3) at 0/3/6/9 dpi; S-0/3/6/9, susceptible genotype (Shannong 20) at 0/3/6/9 dpi.

The stability and repeatability of the UPLC-QE-MS was exhibited in Supplementary Figure 1. Good separation of the wheat metabolites among the R-0 R-3 R-6 R-9, S-0 S-3 S-6 S-9, and QC which were achieved in principle components analysis (PCA) score plots (Figure 3A). This result indicated that FHB stress and wheat genotypes significantly influenced metabolite variation. As shown in Figure 3B, partial least square discriminant analysis (PLS-DA) was conducted to compare the differences between groups R-0 vs. S-0, R-3 vs. S-3, R-6 vs. S-9, and R-9 vs. S-9. The scores plot between PC1 and PC2 in PCA revealed two distinct groups associated with the Sumai 3 and Shannong 20 samples after *F. graminearum* infection, suggesting the distinction in the metabolite accumulation between two genotypic varieties of wheat. PLS-DA (Figure 3B) and volcano plots (Figure 3C) were used of identifying the important metabolites associated with FHB resistance based on the Variable Importance in the Projection (VIP) score (VIP > 1.0), fold change (FC > 2.0 or FC < 0.5) and *P* value <0.05. For Shannong 20 and Sumai 3, the differential metabolites (DFMs) in *F. graminearum* infection period were slightly different, the common DFMs are shown in Table 2, including L-Serine, betaine, O-Phospho-L-serine, L-Glutamate, L-Histidine, and carnosine. Identification results of DFMs between Sumai3 and Shannong20 in 0, 3, 6, and 9 dpi are shown in Supplementary Table 2.

**Table 2:** Potential biomarkers of physiological metabolism.

**Potential biomarkers**	**RT (min)**	**Formula**	**Mass Da Adduct ions**	**KEGG ID**
L-Serine	1.32	C_3_H_7_NO_3_	[M+H]^+^	C00065
Betaine	1.34	C_5_H_11_NO_2_	[M+H]^+^	C00719
O-Phospho-L-serine	6.32	C_3_H_8_NO_6_P	[M+H]^+^	C01005
L-Glutamate	5.22	C_5_H_9_NO_4_	[M+H]^+^	C00025
L-Histidine	1.02	C_6_H_9_N_3_O_2_	[M+H]^+^	C00135
Carnosine	1.41	C_9_H_14_N_4_O_3_	[M+H]^+^	C00386

**Figure 4:**
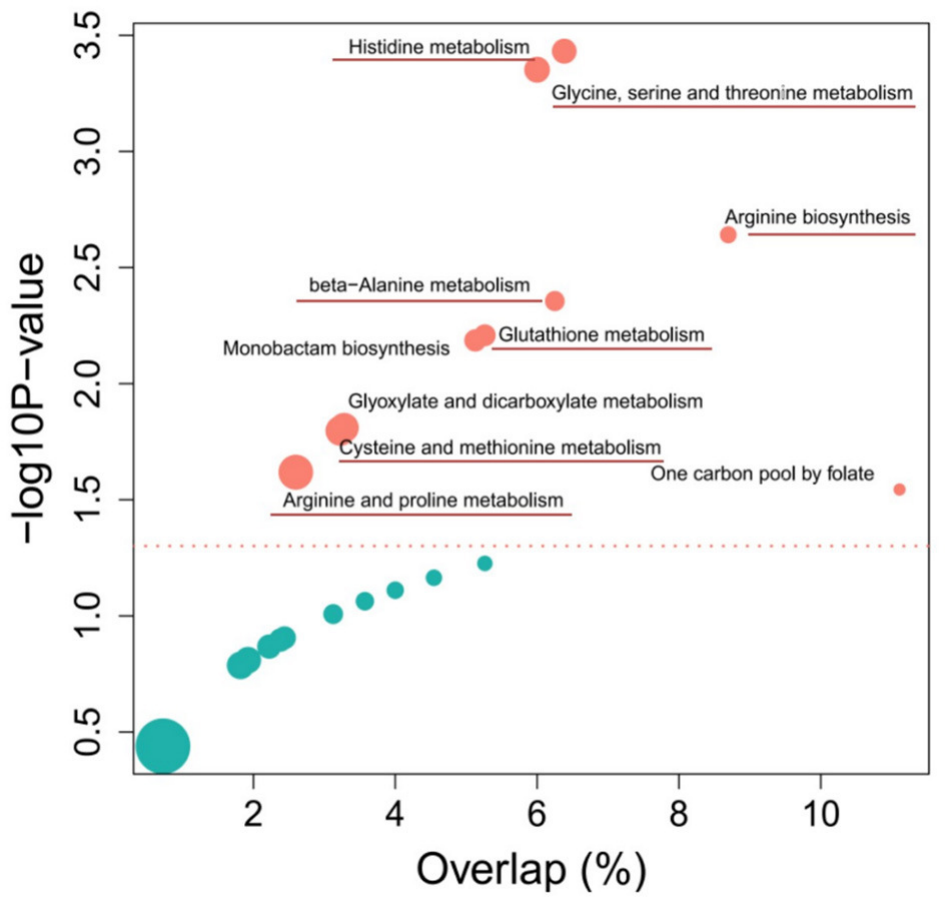
Top 10 enriched metabolic pathways of the comparison between Sumai 3 and Shannong 20 after *F. graminearum* infection. The y-axis and x-axis respectively represent the significance and importance value of the metabolic pathway, bubble size represents the differential metabolites (DFMs) proportion in the metabolic pathway. Detailed information on metabolic pathways in Supplementary Table 3.

The KEGG pathway enrichment analysis from differential metabolites revealed the top 10 pathways that were affected by each of the two different wheat varieties (Figure 4). Among them, most metabolic pathways belong to amino acid metabolism, including histidine metabolism, arginine biosynthesis, beta-Alanine metabolism, cysteine and methionine metabolism, and arginine and proline metabolism pathways. According to the screening of untargeted metabolites and the analysis of metabolic pathways, it was determined that the metabolites with significant differences were enriched in the amino acid pathway. Detailed data on the results of the other metabolic pathway analysis are provided in Supplementary Table 3.

### Absolute Quantitative Analysis of Amino Acids

**Figure 5:**
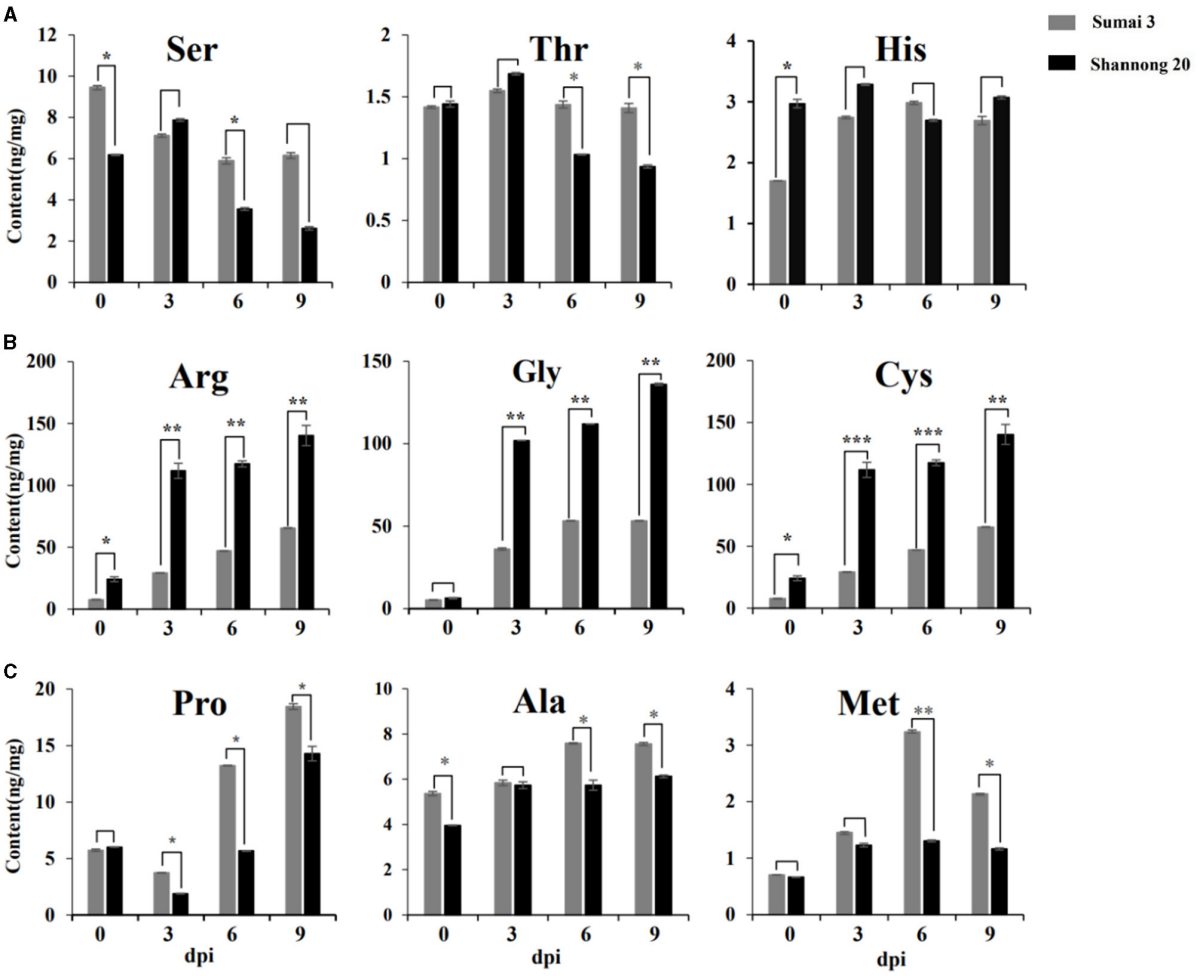
Timeline of amino acids abundances of Sumai 3 and Shannong 20 inoculated *F. graminearum*. Ser, His, and Thr **(A)** showed no obvious rule. Arg, Cys, and Gly **(B)** and Pro, Met, and Ala **(C)** exhibited increased levels in Sumai 3 and Shannong 20 after *F. graminearum* treatment. The contents of Arg, Gly, and Cys were higher (*P* value<0.01) in Shannong 20 than in Sumai 3 at 3, 6, and 9 dpi. The contents of Pro, Ala, and Met were higher (*P* value <0.05) in Sumai 3 than in Shannong 20 at 6 and 9 dpi. Student's *t*-test *P*-value: ***P value<0.001, **P value<0.01, *P value<0.05.

To validate the content of amino acids, targeted metabolomics analysis was performed on Sumai 3 and Shannong 20 samples. Figure 5 exhibits that the amino acid profiles changed a lot between Sumai 3 and Shannong 20 infected with FHB. A total of 9 amino acids accumulated in Sumai 3 and Shannong 20 were subjected to Student's *t*-test. Ser, His, and Thr showed no obvious rule in Sumai 3 or Shannong 20 (Figure 5A). Gly, Met, Arg, Pro, Ala, and Cys showed an increasing trend with the extension of inoculation time in Shannong 20 and Sumai 3 (Figures 5B,C). The contents of Arg, Gly, and Cys were higher (*P* value <0.01) in Shannong 20 than in Sumai 3 at 3, 6, and 9 dpi (Figure 5B). The contents of Pro, Ala, and Met were higher (*P* value <0.05) in Sumai 3 than in Shannong 20 at 6 and 9 dpi (Figure 5C). The results showed that with the days after inoculation of FHB, the levels of amino acids in the two varieties showed different types of changes, and the changes of amino acids might be related to fusarium head blight.

### Exogenous Application Amino Acid Influence Resistance in Wheat

**Figure 6:**
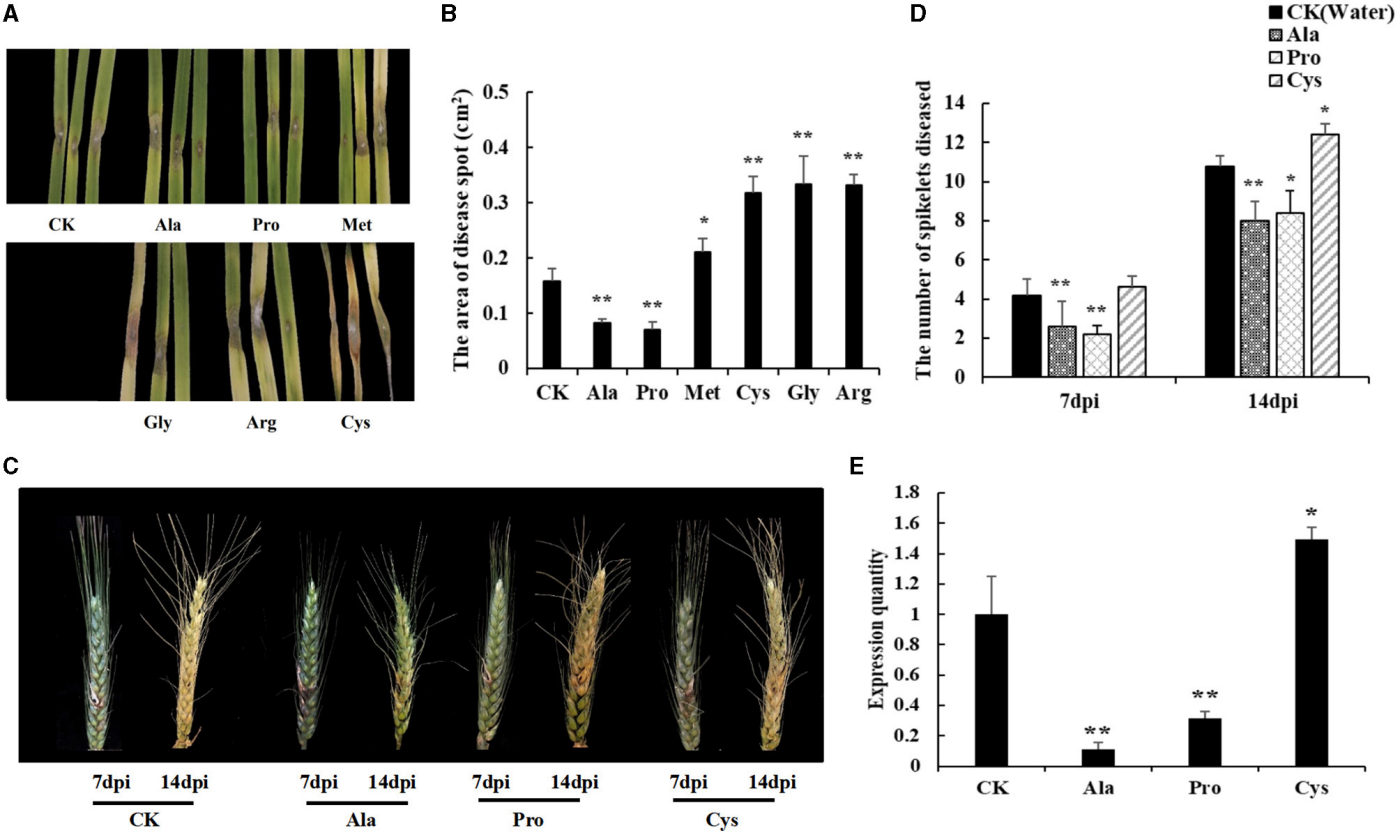
Exogenous amino acid application influences FHB resistance in susceptible genotype wheat. **(A)** The FHB symptoms of detached leaves dipped with an amino acid solution (10mM). Water was used in mock treatments as a control. Pro and Ala treatment can reduce the disease spots on leaves. Cys treatment lead to leaves becoming more seriously diseased. **(B)** The area of disease spot in Shannong 20 detached leaves. Pro and Ala treatment disease spot areas were smaller than in water treatment (*P* value<0.05), Gly, Arg, Cys, and Met treatment increased disease spot on leaves compared to water treatment (*P* value<0.01). **(C)** The FHB symptoms of spikelets sprayed with amino acid solution (10 mM). At 14 dpi, Pro and Ala treatment can significantly reduce the number of diseased spikelets, and lighten the phenomenon of spikelets withered in the upper part of the wheat. **(D)** The number of spikelets diseased. Pro and Ala treatment significantly decreased the treatment at 14 dpi (*P* value<0.05), **(E)** The biomass quantification of *F. graminearum* in infected spikelets. Ala and Pro were significantly reduced compared to CK (*P* value<0.05), Cys was increased the *F. graminearum* relative amount (*P* value<0.05). Student *t*-test *P* value: **P value<0.01, *P value<0.05.

To test the effect of different amino acids (Pro, Ala, Gly, Met, Arg, and Cys) on disease resistance, Shannong 20 detached leaves were immersed into 10 mM amino acid solutions for 24 h, and *F. graminearum* was inoculated into to the detached leaves. Then, the symptoms were observed after 3 days. As shown in Figures 6A,B, Pro and Ala treatment reduced necrotic patches on leaves (*P* value <0.05), while Gly, Arg, Cys and Met increased the wheat sensitivity of *F. graminearum* (*P* value <0.01).

The resistance of different amino acids to *F. graminearum* was also shown in wheat spikelets (Figure 6C). The results showed that Pro and Ala treatments could significantly reduce the number of diseased spikelets (*P* value <0.01), while Cys treatments could aggravate the course of FHB (*P* value <0.05) at 14 dpi (Figure 6D). The biomass quantification showed the relative amount of *F. graminearum* DNA in spikes with Ala and Pro was significantly reduced compared to CK, while that with Cys was increased (Figure 6E). These results also indicated that Pro and Ala played an important role in wheat FHB resistance.

## Discussion

One of the most important fungal diseases of wheat and other cereals in the world is *Fusarium* Head Blight caused mainly by the fungal pathogen *Fusarium graminearum*. It is a worldwide problem to select wheat varieties resistant to FHB because the currently deployed resistance genes often do not confer adequate protection against the accumulation of damaging mycotoxins in wheat grain. Today, there are major challenges to breeding more disease-resistant wheat cultivars.

Plant metabolites continuously responding to biotic and abiotic stress. It is crucial to characterize the metabolome of plants to understand how crops function and respond to stress conditions, produce quality products, learn how to engineer metabolic pathways, and how best to assess food safety. Therefore, in order to increase the understanding of wheat FHB resistance mechanisms, and find metabolites related to FHB resistance, this study used metabolomics analysis to dynamic changes of metabolites from two wheat varieties of FHB inoculation. The result showed that the difference of metabolic pathways was mainly reflected in amino acid metabolic pathways, and selected several amino acids related to FHB resistance (Figures 4, 5). In order to confirm the role of amino acids, exogenous amino acids were applied to the leave and spike of wheat, only Pro and Ala could inhibit the occurrence of *F. graminearum* to a certain extent and significantly improve the resistance of *F. graminearum* in susceptible cultivars, suggesting that Pro and Ala signaling pathways play an important role in wheat resistance to FHB (Figure 6).

Amino acid metabolism and biosynthesis pathway take part in resistance to abiotic or biological stress, regulation of plant growth and development, etc. Recently, the role of amino acids in plant disease resistance has been increasingly researched (Kadotani et al., 2016). Amino acids in plants are involved in primary and secondary metabolism and participate in a wide range of cellular enzymatic reactions as constituents of different enzymes such as aminotransferases, dehydrogenases, lyases, and decarboxylases (Teixeira et al., 2017).

Pro represents a compatible osmolyte, significantly increasing during the stress response in several plants to protect subcellular structures and macro-molecules, which acts as a compatible osmolyte during stress, and was adjusted by balancing its synthesis and catabolism both of which were induced both during and after stress treatments (Parre et al., 2007; Häusler et al., 2014; Batista-Silva et al., 2019). In *Arabidopsis thaliana*, amino acids contents were significantly increased after pathogen infection. Overexpressing some genes (Pro deaminase, PRODH, key enzymes that catalyze proline metabolism) in *A. thaliana* caused an obvious increase in the resistance to pathogen infection (Ward et al., 2010; Cecchini et al., 2011). In addition, Pro provides energy for plant growth, which has distinct protective functions in mitochondria (Welchen et al., 2021). Low doses of exogenous proline could protect plants from salinity, drought, heavy metal, and temperature stress (Teixeira et al., 2017). Similarly, exogenous application Pro to spike and *in vitro* leaves showed resistance to FHB (Figure 6). Thus, the current study indicated that suitable Pro concentration might contribute to the FHB resistance of wheat.

Ala has previously been identified as a precursor in mycotoxin formation by *Fusarium* spp., which was considered as associating with increased susceptibility to disease (Browne, 2007). Nevertheless, in this study, the Ala content in resistant variety Sumai 3 was significantly higher than that in susceptible variety Shannong 20 (Figure 5), and the application of Ala can significantly improve the resistance of wheat to FHB (Figure 6). Ala is synthesized from Glutamic acid, and its transamination with oxoglutarate produces glutamate and pyruvate, a reversible reaction, granting this amino acid a dual function between carbon and nitrogen metabolism (Kendziorek et al., 2012). Ala plays an important role in storing and transferring amino groups under abiotic stress (Ullah and Lim, 2017). Moreover, Ala serves as a precursor of glucosinolates, and glucosinolates play a role in disease resistance (Halkier and Gershenzon, 2006). Therefore, Ala accumulation might be associated with precursors storage for protein synthesis to prepare for rapid recovery of plant metabolism following stress (Nostadt et al., 2020).

The content of cysteine in the susceptible cultivar Shannong 20 was higher than that in resistant cultivar Sumai 3, and increased with the increase of the infection time (Figure 5). Subsequently, exogenous application of cysteine wheat to demonstrated more susceptibility to FHB (Figure 6). A consistent result was found in previous studies, Cys is reactive and toxic when its accumulation exceeds a certain level (Hildebrandt et al., 2015). DON treatment triggered a strong upregulation of cysteine biosynthesis suggesting that cysteine is used for glutathione formation and conjugation to DON (Gardiner et al., 2010). Therefore, silencing genes related to Cys synthesis might improve the susceptibility of FHB, which is the idea of cultivating new varieties.

In summary, we selected a few important amino acids for the improvement of wheat resistance to FHB. Pro and Ala can improve wheat resistance to FHB following further validation. This may serve as an osmotic regulator or secondary metabolite precursor metabolite accumulated in plants to reduce damage to biological stress. In addition, since amino acids are metabolites produced by plants, exogenous application does not pollute the environment. It provides a method for preventing FHB safely in wheat production. Cysteine significantly increases susceptibility to FHB, it can be utilized through repressing candidate amino acids' pathway genes by molecular biological methods to enhance resistance FHB.

## Conclusion

This paper optimized the extraction method of untargeted metabolomics and described the altered metabolic signature of two wheat genotypes (Sumai 3 and Shannong 20) after *F. graminearum* inoculation. The newly established LC–MS-based untargeted and targeted metabolomics allows the investigation of the wheat metabolism and will be an effective tool for other plant studies. Our study indicated that the main altered metabolic pathways in two wheat genotypes after *F. graminearum* inoculation were amino acid metabolisms. Significantly increased amino acids levels were detected and might be related to wheat FHB resistance. In the subsequent experiment in wheat detached leaves by *F. graminearum* inoculation, exogenous Pro and Ala addition increased wheat FHB resistance, while Cys addition aggravates wheat FHB susceptibility. Based on the obtained results, we suppose it can be utilized through stacking or repressing of candidate amino acid pathway genes, to enhance resistance in wheat against FHB. The metabolome data were readily acquired by using UPLC-QE-MS, which was potentially used in a complementary way to QTL in marker-assisted plant breeding programs designed to improve FHB resistance in wheat.

## Data Availability Statement

The original contributions presented in the study are included in the article/Supplementary Material, further inquiries can be directed to the corresponding authors.

## Author Contributions

QX and QW designed the experiments and provided the funding for this research. PZ, SG, YL, and CM performed the experiments and analyzed the data. PZ, SG, and YL performed the LC-MS experiments and analyzed the data. QX, QW, and CH wrote the manuscript. ZD, JB, and JT participated in field tests or helped with the wheat materials. All authors contributed to the article and approved the submitted version.

## Funding

This work was supported by grants from the Major Basic Research Program of Shandong Natural Science Foundation (ZR2019ZD15), National Natural Science Foundation of China (31801330), and Key R&D projects in Shandong Province (2019GNC106060 and 2019JZZY020608).

## Conflict of Interest

The authors declare that the research was conducted in the absence of any commercial or financial relationships that could be construed as a potential conflict of interest.

## Publisher's Note

All claims expressed in this article are solely those of the authors and do not necessarily represent those of their affiliated organizations, or those of the publisher, the editors and the reviewers. Any product that may be evaluated in this article, or claim that may be made by its manufacturer, is not guaranteed or endorsed by the publisher.
