# Supplementary material for: Targeted and Untargeted Metabolomics Profiling of Wheat Reveals Amino Acids Increase Resistance to Fusarium Head Blight
Source: Front Plant Sci. 2021 Nov 19;12:762605. doi: 10.3389/fpls.2021.762605 (PMC8639535; doi:10.3389/fpls.2021.762605)
Supplement: Supplementary file 1 [file Presentation_1.pdf]

Supplementary Figure

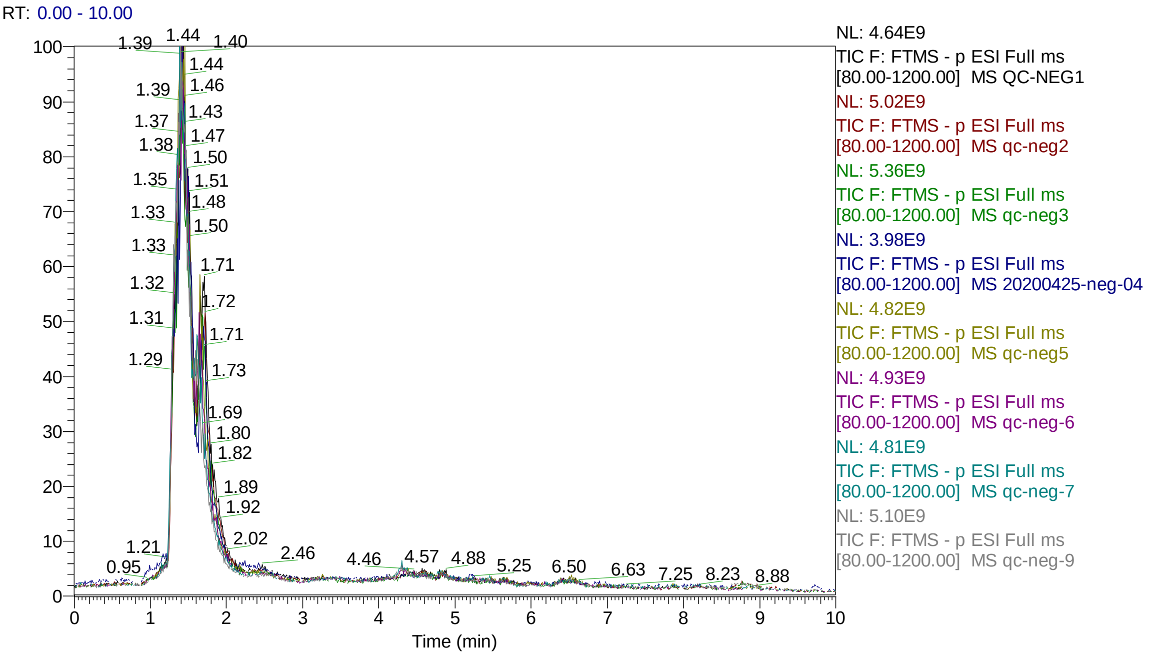

ESI (-)

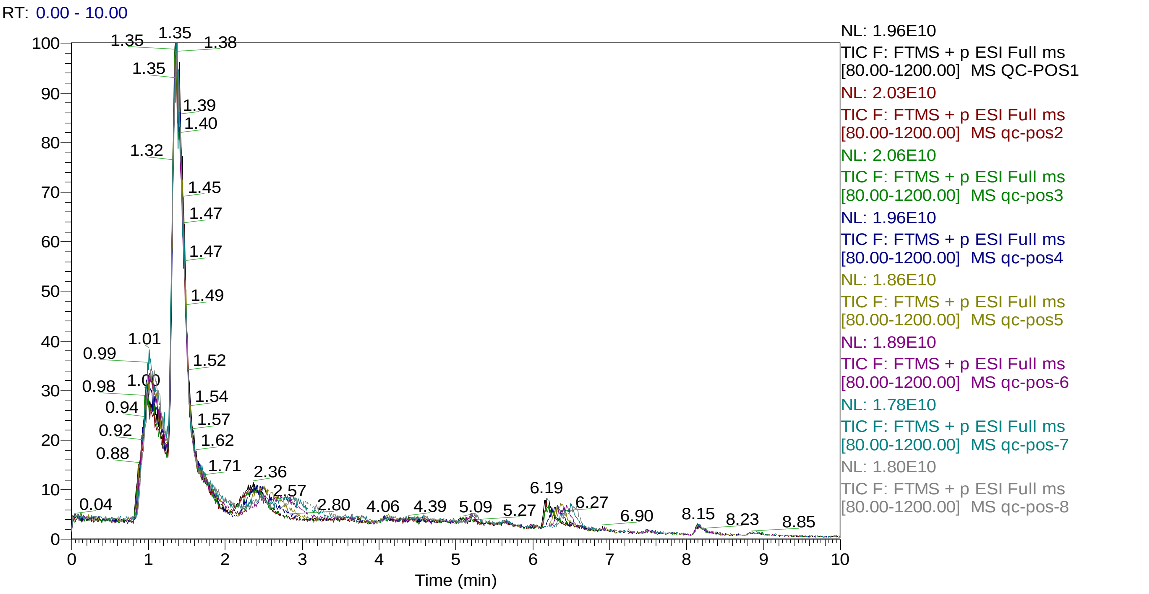

ESI (+)

Supplementary Figure 1. TIC (Total ion chromatogram) showed that the peak shape reproducibility of QC samples was well, indicating that the system was stable.
